# Short Rotation Intensive Culture of Willow, Spent Mushroom Substrate and Ramial Chipped Wood for Bioremediation of a Contaminated Site Used for Land Farming Activities of a Former Petrochemical Plant

**DOI:** 10.3390/plants10030520

**Published:** 2021-03-10

**Authors:** Maxime Fortin Faubert, Mohamed Hijri, Michel Labrecque

**Affiliations:** 1 Institut de Recherche en biologie végétale, Université de Montréal and Jardin Botanique de Montréal, 4101 Sherbrooke East, Montréal, QC H1X 2B2, Canada; maxime.fortin.faubert@umontreal.ca (M.F.F.); mohamed.hijri@umontreal.ca (M.H.); 2 African Genome Center, Mohammed VI Polytechnic University (UM6P), Lot 660, Hay Moulay Rachid, Ben Guerir 43150, Morocco

**Keywords:** willow, *Salix*, phytoremediation, short rotation intensive culture (SRIC), spent mushroom substrate (SMS), ramial chipped wood (RCW), soil contaminants

## Abstract

The aim of this study was to investigate the bioremediation impacts of willows grown in short rotation intensive culture (SRIC) and supplemented or not with spent mushroom substrate (SMS) and ramial chipped wood (RCW). Results did not show that SMS significantly improved either biomass production or phytoremediation efficiency. After the three growing seasons, RCW-amended *S. miyabeana* accumulated significantly more Zn in the shoots, and greater increases of some PAHs were found in the soil of RCW-amended plots than in the soil of the two other ground cover treatments’ plots. Significantly higher Cd concentrations were found in the shoots of cultivar ‘SX61’. The results suggest that ‘SX61’ have reduced the natural attenuation of C10-C50 that occurred in the unvegetated control plots. The presence of willows also tended to increase the total soil concentrations of PCBs. Furthermore, we found that many contaminant concentrations were subject to seasonal oscillations, showing average increases throughout the whole experimental site after a growing period, while showing significantly different variations, such as lesser increases or even decreases, after a dormant period. These observations suggest that contaminants may have leached or degraded faster in untreated conditions, and conversely to have mobilized towards trees through water flow driven by plant transpiration during growing seasons.

## 1. Introduction

Soil contamination with organic and inorganic pollutants is a widespread problematic around the world. Phytoremediation has been proposed as a cost-effective technique that uses plants and amendments to improve physical, biological and chemical soil properties, with the aim of reducing the risks for the environment and for human health [1,2,3]. Plants’ ability to remediate pollutants effectively differs according to species and cultivars, but also depends on the microbiome composition in the rhizosphere [3,4,5]. Soil properties as well as the nature of contaminants and their bioavailability are important factors that affect the mechanisms and efficiency of phytoremediation [6,7]. Polluted areas can be improved with plants and their associated microorganisms by implementing one or more phytoremediation techniques, which include phytoextraction, phytoaccumulation, phytotransformation, phytostabilization, rhizofiltration, phytovolatilization, phytodegradation and rhizodegradation [3,6,8,9]. These plant-based remediation processes are lengthy compared to conventional approaches and the results are difficult to predict, requiring refinement to increase their efficiency. Nonetheless, phytoremediation is considered a safe remediation tool that has been attracting increasing interest and gaining social acceptability over the past 20 years [1,2].

For successful phytoremediation, the plants selected need to be able to survive under the climate in the geographic region of a given site, and be well adapted to its edaphic conditions. Moreover, their tolerance to harsh conditions, such as contamination, pests and other stressors on the site must also be considered [2]. To date, several hundred plants have been studied around the world under different contaminated conditions, enabling identification of many herbaceous and woody plants able to improve the condition of contaminated sites [3,10,11,12,13,14,15].

The facility of willows (*Salix* spp.) and poplars (*Populus* spp.) in adapting to new environmental conditions, combined with their ability to rapidly produce high biomass yield as well as deep, dense roots, make them particularly interesting candidates for phytoremediation of contaminated lands [6,16,17]. Many *Salix* spp. and *Populus* spp. have proven their efficacy in situ for the management of various organic and inorganic contaminants [16,17,18,19,20,21,22,23,24]. Furthermore, the attractive economic value associated to the short rotation intensive culture (SRIC) of these woody plants, especially for bioenergy and biofuel processes, turns phytoremediation using these crops into a promising profitable activity [22,25,26,27]. SRIC of willow and poplar is also increasingly considered as an effective solution to manage urban contaminated brownfields, because the resulting green infrastructures improve the landscape and provide ecosystem services, in addition to contributing to carbon sequestration [16,28,29].

Due to a growing interest in the development of woody crops under contaminated conditions, hundreds of *Salix* taxa were tested in many countries in order to compare their respective performance under different climates [10,22,30,31,32,33]. In Quebec (Canada), several studies conducted on agricultural land, abandoned farmland or contaminated land, have shown that ‘SX61’ and ‘SX64’ (*Salix miyabeana*) are two of the most biomass productive willow cultivars that thrive in eastern Canada [34,35,36,37,38]. Agronomic techniques that promote growth and tolerance of fast-growing species, and increase the mobility and bioavailability of contaminants, are of particular growing interest, because they could leads to overall better remediation [2,3]. Accordingly, several studies focused on inoculation with bacteria and both arbuscular mycorrhizal (AM) fungi and ectomycorrhizal (EM) fungi to enhance the phytoremediation efficacy of willows [8,22,37,39]. Organic soil amendments can also be employed to improve the ability of plants to remediate their environment. In recent years, the use of organic by-products as fertilizer or directly for the remediation of degraded areas has been the focus of several studies [3,40,41,42].

Spent mushroom substrates (SMS) are composted organic wastes, containing agronomic residues and active mycelia, which are a by-product of edible mushroom cultivation. SMS of *Pleurotus* spp. has been studied for potential use as a value-added product, especially as natural organic amendment in soils [43,44] and as an available and cheap source of enzymes for bioremediation purposes [45,46,47]. *Pleurotus* spp. are among the white-rot fungi known for their abilities to degrade organic compounds through the production and secretion of ligninolytic enzymes such as lignin peroxidases, manganese peroxidases and laccases [48]. Ramial chipped wood (RCW) is a substrate made from branches, twigs and leaves, that can support the growth of white-rot fungi, which are the only known organisms capable of degrading all components of wood cell walls, including lignin [49,50,51]. Fungal wood decay processes play important functions in the humic system, and perform better when fungi are associated with other soil microorganisms (i.e., bacteria, algae and protozoa) [52]. The organic soil components formed during RCW-decomposition processes are known to influence the physical, chemical and biological properties of soils, promoting the formation and maintenance of a fertile environment, by improving the water retention capacity and fixing nutrients or making them more available for plants [49,51]. Because of its decomposition-derived organic compounds, RCW has been investigated as a possible amendment for use in the phytoremediation of soils contaminated by trace elements [53,54].

This study was conducted on a contaminated site which has historically (1972 to 1979) been used for land farming activities, specifically, for ex situ waste treatment of a site formerly occupied by a petrochemical refinery plant, located near Montreal (QC, Canada). The refinery’s activities led to the accumulation of moderate to high concentrations of trace elements (TEs), polychlorinated biphenyls (PCBs), aliphatic compounds C10-C50 and polycyclic aromatic hydrocarbons (PAHs) in the soil. More recently (in 2010), two *Salix miyabeana* cultivars (‘SX61’ and ‘SX64’), were successfully established on the site for remediation purposes, following a SRIC technique [55]. Over the initial years of growth, no significant decrease in soil contaminant concentration was reported for any of the compounds tested (unpublished data). The aim of this study was to investigate the effect of two different organic soil amendments (SMS of *Pleurotus ostreatus* and RCW of *Salix* spp.), introduced in this willow plantation, on the bioremediation of TEs, PCBs, C10-C50 and PAHs present in the soil. 

## 2. Results

### 2.1. Initial Soil Contaminant Concentrations (T0)

The initial soil samples (T0) revealed that PCBs, C10-C50, anthracene, benz[a]anthracene, Cr, Cu and Ni were the most problematic contaminants on the site, based on the provincial Land Protection and Rehabilitation Regulation, RLRQ, c. Q-2, r. 37, Sch. I (Table 1). 

Data of Pb and some PAHs were removed from results due to concentrations below levels of concern (data not shown). All other targeted organic and inorganic contaminants have been found at much higher concentrations near plots planted with cultivar ‘SX61’ in the following order: ‘SX61’ > ‘SX64’ ≥ Ctrl. All contaminant concentrations found in ‘SX61’ plots were significantly different (*p* ≤ 0.05) from those found in ‘SX64’ and in Ctrl plots. Significant differences between ‘SX64’ and Ctrl plots were observed for the concentrations of Cr, Ni and Zn only.

The experimental design did not allow us to statistically compare values found at T0 with those observed in the 2010 soil characterization (see Table 6 below). However, visual distributions are shown in the box plots of Figure S1. Soil samples from ‘SX61’ plots generally showed higher mean values of contaminant concentration than those observed during soil characterization. Conversely, the samples collected in the unplanted plots (Ctrl) or in ‘SX64’ plots at T0 generally showed lower or similar values compared to those observed during soil characterization.

### 2.2. Intermediate Variation (IV) and Global Variation (GV) of Soil Contaminant Concentrations

Given the significant differences in soil contaminant concentrations observed between planted and unplanted plots in the initial soil samples (T0), we calculated the IV to compare the treatment effects over time. The mean contaminant concentrations per treatment, as well as their mean IV, are presented for each sampling time in Table S1. Values for T5 can also be considered to be the GV observed over the course of this study. GV of eight compounds (i.e., PCBs, C10-C50 and six PAHs) are presented in Table 2. The IV of contaminant concentrations in soil was not significantly affected by treatments until the third growing season. Indeed, at T4, ground cover treatments showed an effect on the IV_T4_ of 1,3-dimethylnaphthalene. Its variations observed in the RCW plots were significantly different from those observed in the BG plots, with respective values of +52.66% vs. −33.49%. At the end of the third growing season (T5), GV of 1,3-dimethylnaphthalene remained significantly different between RCW plots and BG plots, with respective variations of +45.70% vs. +8.71% (Table 2). The GV of other PAHs, such as benzo[ghi]perylene, naphthalene and 1-methylnaphthalene, also showed significant differences between RCW plots and BG plots, with respective values of +68.66% vs. +16.89%, +31.06% vs. −25.83% and +36.60% vs. −18.58% (Table 2). The GV of 1-methylnaphthalene observed in the RCW (+36.60%) was also significantly different from its GV observed in the RCW+SMS plots (−6.81%). Over the course of this experiment, no significant effect of cultivar treatments was found concerning the IV of TEs, PCBs or PAHs in soil (Table S1). However, after the third growing season, GV of C10-C50 were significantly different between the Ctrl plots and the ‘SX61’ plots, with respective values of −61.55% and −41.86% (Table 2).

### 2.3. Variation Rate (VR) of Soil Contaminant Concentrations

The VR was also calculated to compare the treatment effects over time in a different way. The mean contaminant concentrations and their mean VR per treatment are presented for each sampling time in Table S2. Period type was added as a new two-level variable (growing and dormant) in the VR dataset. The mean VR per treatment was then calculated for each period type and referred to as the mean seasonal variation (mean SV_p_). Table 3 presents the mean SV_p_ of ten contaminants (i.e., PCBs, nickel and eight PAHs), as well as the significant differences, according to the three-way ANOVA between VR values.

In general, PCBs concentrations significantly increased between two consecutive sampling times, moreso in ‘SX64’ than under Ctrl plots, with average VR of +17.03% vs +11.37%, respectively. The concentrations of benz[a]anthracene, benzo[ghi]perylene, chrysene, naphthalene, phenanthrene, 1-methylnaphthalene and 1,3-dimethylnaphthalene increased significantly more under RCW plots than under either one or both of the other ground cover treatments (BG and RCW+SMS) (Table 3). The period type had a significant effect on the variation rates of PCBs, Cd, Ni and ten (10) PAHs concentrations in soil. The positive VR of PCBs, Cd, Ni, anthracene, benzo[ghi]perylene and chrysene observed after a growing period, was significantly different from their negative VR observed after a dormant period. Fluoranthene, indeno[1,2,3-cd]pyrene, phenanthrene and pyrene, showed positive VR after both periods, but their mean VR after a growing one was significantly higher than observed after a dormant period. Naphthalene, 1-methylnaphthalene and 2,3,5-trimethylnaphthalene also showed positive VR after both periods, but a reverse pattern with significantly greater increases after a dormant period, than after a growing one. Values concerning SV_p_ of all contaminants, including Cd, anthracene, fluoranthene, indeno[1,2,3-cd]pyrene and pyrene, can be found in Table S3.

**Figure 1:**
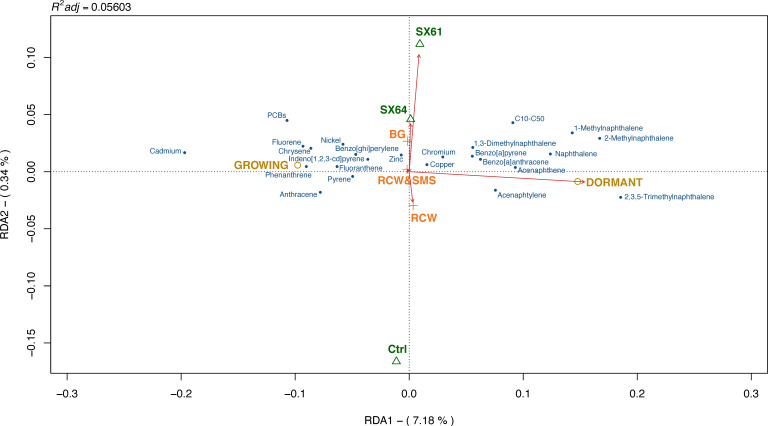
Redundancy analysis (RDA) showing the relationship between treatments, periods and the variation rates (VR) of contaminants in soil. Blue labels represent the VR of each “contaminant”. Green open triangles, yellow open circles and orange cross symbol represent “factor centroids” of environmental variables. Red-line arrows represent the “biplot scores” of environmental variables. The length of each arrow indicates the contribution of the corresponding variable to the contaminant variation rates.

Redundancy analysis (RDA) was conducted to evaluate the relationship between soil contaminant VR and ground cover treatments (BG, RCW or RCW+SMS), cultivar treatments (Ctrl, ‘SX61’ or ‘SX64’), as well as periods (growing or dormant) (Figure 1). According to the permutation test, only period variables had significant impact on soil contaminant variations (r^2^ = 0.06708, *p* ≤ 0.001 ***), followed by cultivar treatments (r^2^ = −0.00529, *p* = 0.972) and ground cover treatments (r^2^ = −0.00683, *p* = 1.00). Only the first RDA axis was significant (*p* ≤ 0.001 ***), explaining 7.18% of the cumulative percentage variance of all soil contaminant variations. The entire RDA model was significant (*p* ≤ 0.001 ***).

### 2.4. Water Extracted TEs, pH and EC

The water extracted fraction of TEs from soil samples, as well as pH and electrical conductivity (EC), were assessed in the RCW and BG subsections of ‘SX61’ and Ctrl treatments, at T0, T1 and T5 only (Table 4). The limited portion of the site was selected in order to evaluate the impact of willow trees and soil organic amendments on the bioavailability of TE. The ground cover and cultivar treatments (RCW and ‘SX61’) showed no significant effect on pH and EC at any sampling time. Among the six targeted TE, the water extracted concentration of Cu was the highest, followed by Cr, Zn, Ni and Pb (data not shown for Pb), while the water extracted concentrations of Cd were under the detection limit in all soil samples. All four water extracted TEs (i.e., Cr, Cu, Ni and Zn) were found in similar concentrations in ‘SX61’ and Ctrl plots in the initial soil samples (T0). After the first growing season (T1), the water extracted concentration of Cr was significantly higher in ‘SX61’ plots than in Ctrl plots, but only under the BG treatment. Its water extracted concentration was also significantly higher in RCW plots than in BG plots under the Ctrl plots only. After the third growing season (T5), the water extracted concentration of Cr rose to significantly higher levels in all RCW plots compared to BG plots and also rose to significantly higher levels in all ‘SX61’ plots compared to all Ctrl plots. The Cu water extracted concentration was significantly higher in ‘SX61’ plots than in Ctrl plots, after the first and third growing seasons (T1 and T5). The water extracted concentration of Zn followed the same trend as Cr and Cu, but the differences between ‘SX61’ and Ctrl plots were significant only after the third growing season (T5). Conversely, the water extracted concentration of Ni showed significantly higher values in Ctrl than in ‘SX61’ plots, after the first and third growing seasons (T1 and T5).

### 2.5. Biomass Production and TE Phytoextraction

Willow biomass parameters measured after the third growing season (T5) are presented in Table 5. The equivalent of 75.3 and 83.6 odt ha^−1^ was harvested for ‘SX61’ and ‘SX64’ respectively. On an annual basis, these values respectively represent yields of 25.1 and 27.9 odt ha^−1^ yr^−1^. The differences between the two cultivars were not significant nor did the ground cover treatments impact biomass yield. Moisture levels inside the willow shoots varied significantly between the cultivars, with 1.94% more water in ‘SX61’ than in ‘SX64’.

No trace of Cr, Ni or Pb was detected in the aerial tissues of the two willow cultivars. Zn was found in the highest concentrations, following by Cu and Cd. Significantly higher Cd concentrations were found in the shoots of ‘SX61’ than in ‘SX64’ shoots (*p* = 0.024). Therefore, no significant difference in the total extracted quantities of Cd was observed between the two willow cultivars. The BCF of Cd were also similar between both cultivars.

The average concentration of Zn in the willow shoots was significantly higher in RCW and RCW+SMS plots compared to BG plots (*p* ≤ 0.01). Consequently, the total quantity of Zn extracted per hectare was significantly greater under RCW and RCW+SMS plots than in BG plots (*p* = 0.027). The Zn BCF was similar between ground cover treatments but was significantly higher in ‘SX64’ than in ‘SX61’ (*p* = 0.028). The Cu concentrations observed in the willow shoots did not vary significantly between cultivars or ground cover treatments, leading to similar potential extraction quantities per ha and year. The BCF of Cu was similar between all treatments.

## 3. Discussion

### 3.1. Biomass Production

Biomass production of both cultivars, reached on a contaminated soil, compare favorably to other results obtained on farmland in southern Quebec [35,36]. In their long-term trial, Labrecque and Teodorescu [35] found no difference in growth between the same two cultivars (‘SX61’ and ‘SX64’) and reported respective annual yields of 20.4 and 23.7 odt ha^−1^ yr^−1^ for the second rotation cycle, and 21.3 and 24.3 odt ha^−1^ yr^−1^ for the third. Their recorded biomass yields were slightly lower than those observed in this study, but their estimates were based on a planting density of 18,000 cuttings per hectare, which is less than the 18,500 here. This high productivity, combined with the absence of visual symptoms of toxicity, together suggest that these two cultivars can be successfully cultivated in moderately contaminated soils under northern climatic conditions such as those found in Quebec. Also, it should be noted that the experimental site was polluted by activities that included spreading contaminated sediments of a decantation basins containing cocktails of organic and inorganic residues (land farming), but over a rich agricultural soil base. Many *Salix* spp. have demonstrated high biomass productivity and tolerance to petroleum hydrocarbons (PHCs) in southern Quebec [34]. Low concentrations of PAHs have even been reported to increase the growth and transpiration rate of some willow trees [56].

Increasing soil organic matter has been reported to have positive effects on soil properties and crop performance [57]. Stem biomass of *Salix viminalis* and *S. discolor* have been found to be significantly greater when receiving wastewater sludge treatment, resulting in productivity exceeding that of control plants by five to seven times [58]. After a three-year growth cycle, *S. dasyclados* also produced significantly greater biomass in organically amended plots than in control plots [59]. Because of this, it was expected that higher biomass yields would be observed after the application of soil organic amendments such as RCW and RCW+SMS. However, observation of plant growth over the three seasons of this study revealed no impact of these amendments on the biomass production of the two willow cultivars. These results may reflect the excellent agronomic conditions of the site as well as its adequate water supply, due to its proximity to the St. Lawrence River (less than 350 m) and low elevation (about 3.5 m above the river water level). Increased plant growth under SMS and RCW is usually attributed to improvement of the soil conditions and nutrients supplied, when the amendments are the sole source of fertilizer, compared to the depleted soils used as controls [60,61,62,63,64].

### 3.2. TE Phytoextraction

Among the six-targeted TEs analyzed in the aboveground tissues, Zn was found in the highest concentration, followed by Cu and Cd, while Cr, Ni and Pb were not detected. These results compare well with previous phytoremediation field trials, which reported similar rankings of TE concentrations in the aerial parts of *Salix* spp., with little or no trace of Cr, Ni and Pb [20,65,66].

The total concentration of TE in soil is an important factor that can influence the rate of phytoextraction [20]. For instance, at the beginning of the present study (T0), total concentrations of Cu and Zn in soil were relatively higher (from 792 to 2381 mg kg^−1^ and from 234 to 479 mg kg^−1^), while Cd and Ni were relatively lower (from 1.8 to 2.2 mg kg^−1^ and from 66.2 to 106.5 mg kg^−1^). This may partly explain why concentrations of Zn and Cu were higher than those of Cd and Ni in willow shoot samples.

Our results may also reflect the presence of H_2_O-soluble TEs in soil. The metals present in the water extract can be considered as the fraction most readily bioavailable for plant uptake and are generally well correlated with phytoextraction [67]. The extent of elemental partitioning between the aqueous and solid phases in soils is very dynamic and depends on multiple physical, chemical and biological processes and their interaction. These processes include precipitation-dissolution, adsorption-desorption, complexation and encapsulation, which are mainly moderated by factors such as the nature of the element and their speciation, the structure and penetrability of soil, pH, electrical conductivity (EC), cation-exchange capacity (CEC) and percent of organic matter and dissolved organic carbon (DOC) [68,69,70,71]. It is also well known that total soil TE content largely determines the partitioning of TE between the solid and solution phases [68,69]. Consequently, the water extracted concentrations of all TEs followed the same order as their total concentrations in soil: Cu > Cr > Zn > Ni > Cd.

Although chromium was the second most abundant and bioavailable TE in soil, it did not seem to have been taken up by the plants to the same extent as other TEs, which highlights that phytoextraction is highly metal specific. Chromium is generally minimally translocated within plants [55,71,72] and tends to be concentrated mainly in roots, apparently because of its propensity to bind to cell walls [73].

In this experiment, Cd is the only element for which a BCF greater than 1 was calculated. BCFs for other TEs were rather low (from 0.14 to 0.26 for Zn and 0.0021 to 0.0056 for Cu). In a field experiment, Kacálková et al. [74] found the BCF of Cd and Zn to be greater than 1 in *Salix smithiana* and *S. rubens*, and lower than 1 for Cu. Our results also compared well with those of Pitre et al. [75], who reported a Zn BCF of 0.23 in *S. miyabeana* during a brownfield trial. A low Pb and Cu BCF, varying from 0.05 to 0.15, and a higher BCF for Zn and Cd, ranging between 2.0 and 7.0, were also reported in a study conduct on a military landfill [20]. During a pot experiment, Desjardins et al. [76] found a slightly higher BCF for Zn (0.68), with higher concentrations in aerial tissues of *S. miyabeana* (119.96 mg kg^−1^) compared to those reported here.

Willow species and cultivars may differ in their ability to translocate TEs from roots to shoots [65,77]. In this study, significantly higher Cd concentrations were found in the shoots of ‘SX61’ than in ‘SX64’. However, the quantities of Cd (ha^−1^ yr^−1^) extracted were statistically similar. Extrapolating the amounts of TEs phytoextracted per unit of area represents a useful exercise to estimate the phytoextraction potential of various plants. Both cultivars showed similar concentrations of Zn and Cu in their aerial tissues, which may suggest similar extraction capacity for these TE, and greater Cd extraction capacity in ‘SX61’. However, it is difficult to compare their phytoextraction efficacy, because the means of all TEs concentrations in soil were significantly higher in ‘SX61’ plots than in ‘SX64’ plots at the beginning of the study (T0). In view of this, it is reasonable to suggest that ‘SX64’ has a greater extraction capacity than ‘SX61’ for Zn, Cu and Cd, since the BCF of Zn was significantly higher in ‘SX64’ than in ‘SX61’ and the BCF of Cu and Cd showed a tendency to be higher under ‘SX64’ than in ‘SX61’ (*p* = 0.079 and *p* = 0.089). Although there is no significant differences in phytoextraction efficacy between the two cultivars, our results agree with the finding that *Salix* spp. are more suitable for the phytoextraction of Cd and Zn than Cu and Cr [72,78,79].

### 3.3. Soil Organic Amendment and TE Phytoextraction

Assessing the impact of soil amendments on willow phytoextraction efficiency revealed that the SMS did not affect TE phytoextraction in either willow cultivar. A reduction in the extraction rate was expected, since Gąsecka et al. [80] found that content of Cu in roots, leaves and shoots of *Salix purpurea × viminalis* hybrid was reduced by the addition of *Pleurotus ostreatus* SMS. Although it differs in composition, spent mushroom compost (SMC) from the production of *Agaricus bisporus* was also effective in improving Cu phytostabilization by the same *Salix* hybrid in another hydroponic experiment [81]. However, in small doses (addition of 10% SMC to 90% sand, instead of 30% SMC to 70% sand), it has been reported to have stimulated plant growth and efficiency of Cu accumulation of the same hybrid willow [81]. In a greenhouse experiment carried out in plastic pots, Frutos et al. [61] found that a mixture of SMC of *A. bisporus* and SMS of *P. ostreatus* reduced Cu, Cd and Pb translocation to the shoots of *Atriplex halimus*. They attributed their results to a progressive decrease of water-soluble fractions of TEs in the soil with increasing doses of SMC/SMS. They also found that roots of *A. halimus* accumulated significantly more Cu and Pb with increasing doses of SMC/SMS, despite the decrease of Cu and Pb in the leachate. It would have been interesting to measure TE concentrations in the roots of our willow cultivars to see if similar trends of phytostabilization would have been observed with the addition of SMS of *P. ostreatus*. However, the focus here was on monitoring the accumulation of TE in the shoots only.

In our study, RCW alone or mixed with SMS significantly increased the concentrations of Zn in willow shoots, by 17.27% after three seasons of treatment, suggesting its potential effect on Zn speciation and bioavailability. However, our results did not show any difference in water extracted concentrations of Zn between soil samples collected in BG and RCW plots. Nevertheless, a tendency towards higher dissolved concentrations of Zn (*p =* 0.070) was observed under the RCW plot at T1, once again suggesting a possible impact of RCW on Zn bioavailability. Such changes in Zn bioavailability in soil, combined with rapid uptake and translocation to plant shoots may explain the higher concentrations of Zn in the shoots of trees that grew under RCW alone or mixed with SMS. Simultaneous extractions of Zn may have masked a stronger effect of RCW on Zn bioavailability. Conversely, an exclusionary mechanism for Cr in *Salix* spp. may have led to a stronger effect of RCW on the bioavailability of this TEs in soil.

TEs bioavailability may have been increased by the RCW due to the formation of soluble organo-metallic complexes [82,83]. Nguyen et al. [70] also demonstrated a significant correlation between TEs bioavailability, notably Ni and Cu, and the concentrations of the dissolved organic carbon in soil. Organic amendments, such as composted sewage sludge or fresh RCW, are known to increase humic substances (HS) in soil, which can influence TEs speciation, thereby affecting their mobility and bioavailability [53]. Humic acids (HA) are known to be more insoluble and may contribute to TEs immobilization, while fulvic acids (FA) are more water-soluble and may be responsible for TEs solubilization [84]. In soils, Zn seems to bond predominantly on FA [53,85,86]. The formation of Zn-FA complexes might then explain our phytoextraction results, by making Zn more bioavailable to willows. Hattab et al. [54] reported that applying RCW favours TEs immobilization and reduces their phytoavailability, which differs from our results.

### 3.4. Soil Contaminants in the Initial Soil Samples (T0)

The initial soil samples (T0) showed greater concentrations of all organic and inorganic contaminants in planted plots (‘SX61’ and ‘SX64’) than in unplanted ones (Ctrl). This finding strongly suggests that the presence of willows on the site during the four years prior to the beginning of the current study influenced the spatial distribution of all contaminants in the soil. This observation seems to lie outside of the remediation objectives that motivated the establishment of this willow plantation on the site by Guidi et al. [55]. Unsuccessful and inconclusive field trials reporting similar patterns with organic contaminant, have usually attributed such results to lower degradative microorganism activity in the rhizosphere [87,88]. However, because inorganics cannot be degraded, such explanations cannot account for the finding that TEs concentrations followed the same trend as organic compounds in the initial soil samples. Rather, this suggest that another phenomenon, such as soil contaminant migrations, may also have contributed to driving the differences in total contaminant concentrations in the soil.

Because most contaminants tended to be more concentrated under ‘SX61’ and less or similarly concentrated under Ctrl plots, than levels measured four years before (Figure S1), such differences may either have been the result of greater decreases in the unplanted plots, greater increases in the planted plots or even a combination of both. Unfortunately, it was impossible to determine with certainty which of these phenomena would more likely explain the observed pattern, since our experimental design did not allow us to statistically compare values between T0 and those from the 2010 soil characterization.

### 3.5. Willows and Global Variations (GV) of Contaminants in Soil

At the end of the present study, only GV of C10-C50 was significantly affected by the presence of willows. The observed differences in its GV suggest that cultivar ‘SX61’ reduced what could be described as the “natural attenuation” occurring in the unvegetated plots (Ctrl). The smaller decrease of C10-C50 in the ‘SX61’ plots align with the proposed explanations concerning its greater concentrations in planted plots than in unplanted ones previously observed in our initial soil samples (T0). However, it is difficult to determine if these smaller decreases were the result of less degradation or, alternatively, the result of less leaching.

The experimental site was an open system allowing vertical and/or horizontal water movement, which may have impacted contaminant migration in soil, as suggested in other studies [89,90]. The willow plantation may have acted as a vegetated cap, preventing or minimizing leachate of contaminants, which is an important part of the natural attenuation process [91]. The foliage and canopy cover can physically slow down rainfall, thereby minimizing water infiltration into the soil. Additionally, willows are fast growing phreatophytic woody plants with a high transpiration rate, making them strong biological pumps for ground water in cool temperate regions [92]. When exploited as evapotranspiration cover in SRIC, they can strongly influence soil hydrological dynamics [93] and reduce the deep percolation and leaching of toxic products into the environment [94,95,96,97]. Such impacts on soil hydrological dynamics may have contributed to the smaller reductions of C10-C50 observed under ‘SX61’.

Our results revealed that the GV of all other contaminants was not statistically influenced by the willow plantation. However, we suspect that the GV of PCBs may also have been affected, to a lesser extent, by the presence of willows. The final concentrations of PCBs determined for Ctrl plots were, on average, +1.66% higher than those at T0, while they were found to be, on average, +21.96% and +38.13% higher in ‘SX61’ and ‘SX64’ plots, respectively. These GV were not significantly different between treatment (*p* = 0.081), possibly because of the high variability in our dataset. The standard deviations of the results well reflect the inherent heterogeneous distribution of pollutants on the whole experimental site. To overcome the methodological problems associated with such heterogeneity, a value of 10% (*p* ≤ 0.1), instead of 5% (*p* ≤ 0.05), has been proposed as an acceptable level of significance in field studies [98]. Based on such a standard, the presence of willows can be considered to have increased the total soil concentrations of PCBs over the course of our study.

By removing a substantial amount of water from the groundwater, willows can increase the flux of dissolved contaminants from the water capture zone towards the rooting zone [92]. There is evidence that some plants can impact the movement of organic compounds such as PAHs, leading to their accumulation in the rhizosphere [99]. Similarly, elevated concentrations of Pb have been observed in the rooting zone of *Betula occidentalis* growing on contaminated soil collected from an abandoned mining site in Utah [100]. While, to our knowledge, this phenomenon has never been reported for PCBs, it may be a possible explanation for the trend in PCBs GV observed here. Such contaminant supply by migration could lead to apparent increases in concentrations over time, even if degradation and/or phytoextraction are actively occurring [98,100].

Our results show that considerable amounts of Zn, Cu and Cd were removed from the ground by phytoextraction. However, their GV in soil were not significantly different between the planted (‘SX61’ and ‘SX64’) and unplanted plots (Ctrl) after the duration of this study. The highly heterogeneous distribution of pollutants on the site may have masked significant results here, too. Simultaneous transfers, by water mass flow or diffusion toward the root zones, gradually reloading the soil in TEs in parallel to extraction, may also have contributed to mask significant results. The nature and intensity of changes in contaminant concentrations in the root zones may thus depend on the correspondence of contaminant supply by the adjacent bulk soil, and its elimination through plant uptake and/or by degradation. Consequently, it would be very difficult to monitor pollutant variations in the field by sampling surface soil that is extensively penetrated by roots [99]. Total plant uptake could be assessed as a measure of success in the field, rather than attempting to quantify decreases in soil contamination at the end of a growing season [98].

### 3.6. Variation Rates (VR)

Monitoring soil contaminant variation according to the previous sampling time, revealed that many contaminant concentrations were subject to seasonal oscillations. Almost all contaminants (except C10-C50) showed average increases throughout the whole experimental site after a growing period, while many showed significantly different VR, such as lower average increases (i.e., fluoranthene, indeno[1,2,3-cd]pyrene, phenanthrene and pyrene), or even average decreases (i.e., PCBs, Cd, Ni, anthracene, benzo[ghi]perylene and chrysene), after a dormant period.

Because deciduous trees are dormant for part of the year, the impact of vegetation on water movement and contaminant mobilization (or immobilization) would be more likely to occur over a growing period, when roots are more active, evapotranspiration is maximal and precipitation is low [92,97,101]. Although seasonal oscillations can be assumed to occur over the whole experimental site, including the Ctrl plots, they could still have been driven by the high transpiration rates occurring in the willow plantation throughout the growing seasons. It is important to mention that Ctrl plots were located only a few meters from the plantation (0–3 m), and that no physical barriers were installed to prevent willow roots from developing there over time and pumping a large amount of water into it.

Interestingly, naphthalene, 1-methylnaphthalene and 2,3,5-trimethylnaphthalene were the only contaminants to show the reverse pattern, with significantly higher increases after a dormant period than after a growing one. These two-ring naphthalenes may have increased as a result of migrating from the adjacent bulk soil, or due to degradation of other PAHs containing three or more rings in the molecule, as suspected by Gąsecka et al. [102]. Their study found rapid increases of naphthalene concentrations following the degradation of other PAHs (i.e., anthracene, pyrene, phenanthrene and fluoranthene) by *Agaricus bisporus* and *Lentinula edodes* in a 12-week experiment. These three naphthalenes have a low molecular weight and are among the more water-soluble PAHs (low Log K_ow_), which makes them more susceptible than other PAHs to be taken up by plants or to be degraded. Based on our observations, we agree that sampling protocols for monitoring contaminant variations in field should account for potential seasonal fluctuations and specify that soil samples be compared between similar seasons [92].

### 3.7. Willows and Water-Soluble Fractions in Soil

Willow growth seems to have affected TEs availability in our experimental site, because differences in the water extracted fraction of Cr, Cu, Ni and Zn were observed between ‘SX61’ and Ctrl plots at the end of two different growing seasons (T1 and/or T5). In the scientific literature, changes in labile soil TEs under trees are quite inconsistent. For example, under a cold climate field trial, Courchesne et al. [103] showed that the willow cultivars used (‘Fish Creek’ and ‘SX67’) significantly decreased the total, as well as the labile pool of As, Cd, Cu, Ni, Pb and Zn concentrations in soil, over a three-year period. Conversely, Pulford et al. [65] found that the concentrations of EDTA extractable Cd, Cu, Ni and Zn were significantly higher in soil collected from under willows than from unplanted areas. Nguyen et al. [70] reported that the labile pool of several trace elements was influenced by the presence of plants but varied according to the plant species.

Plant species have a distinct influence on the labile pools of TEs in soil, essentially by controlling pH and organic matter content [67]. Some pH changes can occur in the rhizosphere as a result of the differential uptake rates of cations and anions in plant roots [104]. When the influx of cations is much higher than that of anions, protons are released to compensate for an excess of positive charges. Soil acidification invariably increased solubility of positively charged ions (such as Cd^2+^) due to increased competition from H^+^ ions at the negatively charged binding sites [105]. Conversely, hydroxyl or bicarbonate ions can be released by plants to compensate for an excess of negative charges in roots, increasing sorption and precipitation of TE cations, which decreases their availability in soil [106]. TEs solubility is then generally found to increase markedly under acidic conditions [107]. Plants can also alter the pH in soil with the release of carbon dioxide (CO_2_) following the respiration of root cells and that of their associated microorganisms [108,109]. Additionally, root exudation, which includes several forms of carbohydrates, proteins, organic acids, amino acids, and phenolic compounds, may affect TE solubility in soil, via direct complexation, or indirectly, by providing energy substrates for rhizosphere microflora, or by affecting pH [70,109].

Results of our experiment indicated that pH values were close to 8 in all samples and remained stable over time. However, pH changes may have occurred very close to willow roots at a given time without being detected in the adjacent bulk soil. Supporting this point, Cd was observed in willow shoots, with BCF values close to 1, even though its water extracted concentrations were under the detection threshold in all soil samples. Apparently, Cd is more soluble in soils within the range of pH 4.5–5.5, but more immobile above a pH of 7.5 [71]. Cd may thus have been solubilized in the rhizosphere and/or in the hyphosphere [110], taken up rapidly and translocated to plant shoots.

It has been proposed that if TEs were not taken up by plant roots, increases in soil TEs availability could lead them to leach into the environment [101,111,112]. We cannot exclude the possibility that such a phenomenon occurred on the study site in the past and may thus have helped drive the differences in their total concentrations in our initial soil samples (T0). However, this does not seem to have occurred during the current study, since the GV in soil concentrations of Cr, Cu and Zn showed no significant differences between treatments, although their water-soluble fractions were higher under planted plots (‘SX61’) than under unplanted ones (Ctrl).

Plants can also impact the dissolved chemical concentrations in the soil solution close to their roots as a result of water and nutrient uptake [113,114]. When elements are found in large concentrations in the soil solution, their enrichment can be expected in the root zones as a consequence of a transfer to the root-soil solution interface by mass flow, at a greater flux than required by the roots [114]. In fact, the water extracted fractions of Cr and Cu were the highest among the six TEs targeted in this study and they showed significantly higher concentrations under planted plots (‘SX61’) than under unplanted ones (Ctrl), after the first (T1) and third growing seasons (T5). The water extracted fractions of Zn were slightly lower than those of Cr and Cu, and only showed significant differences after the third growing season. Conversely, Ni had the lowest water extracted concentrations of the five targeted TE, and showed completely opposite results, suggesting that Ni may have been transferred at a lower flux than it was taken up by plant roots.

Forward diffusion or back-diffusion of the solutes through the concentration gradients can occur, following their depletion or enrichment in the root zones [114]. We believe that such a phenomenon may have occurred throughout our study, thereby tending to balance the water-soluble fractions of TEs throughout the site, more particularly during the dormant periods, as observed in our initial soil samples (T0), collected after the winter months, when deciduous trees had been dormant for a long period.

### 3.8. Organic Amendment and Soil Contaminants Concentrations

Differences in the GV of contaminants were expected following application of SMS to the soil, but none were observed for any contaminant over the three seasons of the study. Soil contamination may have reduced the effectiveness of SMS for soil decontamination. García-Delgado et al. [115] showed that TEs, such as Cd and Pb, inhibited laccases activity of *Pleurotus ostreatus* SMS, thus decreasing the biodegradation rate of PAHs. Here, it is also important to note that the RCW+SMS mixture was applied on the soil surface only, without being mixed deeply into the soil, which may have prevented contact between fungal enzymes and organic contaminants. The growth of *Salix planifolia* in plastic bins containing contaminated soil, previously mixed with woodchip colonized by *P. ostreatus*, led to higher rates of hydrocarbon removal than natural attenuation [116]. Unfortunately, in the latter study, the effect of willow was not evaluated separately. It was therefore impossible to determine whether their amendment improved the performance of the willow in remediating their petroleum-contaminated soil.

Although RCW was mainly used to support the metabolic activities of SMS, its application alone affected the GV of PAHs over time, with significantly higher increases of benzo[g,h,i]perylene, naphthalene, 1-methylnaphthalene and 1,3-dimethylnaphthalene in RCW plots than in BG ones. Interestingly, among these four PAHs, three were two-ring naphthalenes. A high organic content appears to exert strong PAHs sorption capacities in soil with a more pronounced impact on the lighter PAHs, reducing their mobility [90]. Other PAHs (i.e., anthracene, benz[a]anthracene, fluorene, indeno[1,2,3-cd]pyrene, phenanthrene, pyrene and 2-methylnaphthalene) also showed a tendency to follow the same trend (*p* ≤ 0.1), with either greater increases in RCW plots, or greater decreases in BG ones. Although the water extracted fraction of organic contaminants was not assessed in the present study, organic matter resulting from the humification of RCW may have induced the immobilization of some PAHs at the soil surface, resulting in their accumulation over time, after repeated groundwater movement. Incorporating SMS into RCW may have disrupted its degradation rate, thereby reducing its effect on the GV of PAHs.

## 4. Materials and Methods

### 4.1. Experimental Site

The experimental site is a flat area of 5840 m^2^, located in the municipality of Varennes, on the south shore of the St. Lawrence River, across from the Island of Montreal (QC, Canada, 45°42′02.8″ N, 73°25′53.4″ W). The region has a continental climate characterized by an annual average temperature of 6.6 ᵒ C and annual average precipitation of 981 mm [117]. The petrochemical factory (PÉTROMONT INC.) that operated for many years on the site was shut down in 2008. During the period of industrial operation, settling ponds were built, to control liquid discharge from refining processes. Between 1972 and 1979, sludge collected from the bottom of these basins was spread on adjacent land (the current experimental site) according to land farming practices.

The soil on the site was characterized in 2010. Agronomic properties and contaminant concentrations are presented in Table 6. PCBs, Cu, Cr and anthracene were considered to be the most problematic contaminants on the site, based on the provincial Land Protection and Rehabilitation Regulation, RLRQ, c. Q-2, r. 37, Sch. I. These contaminants were found mainly in the surface soil (0–60 cm), as described in Guidi et al. [55].

### 4.2. Experimental Planting and Maintenance of the Plantation

**Figure 2:**
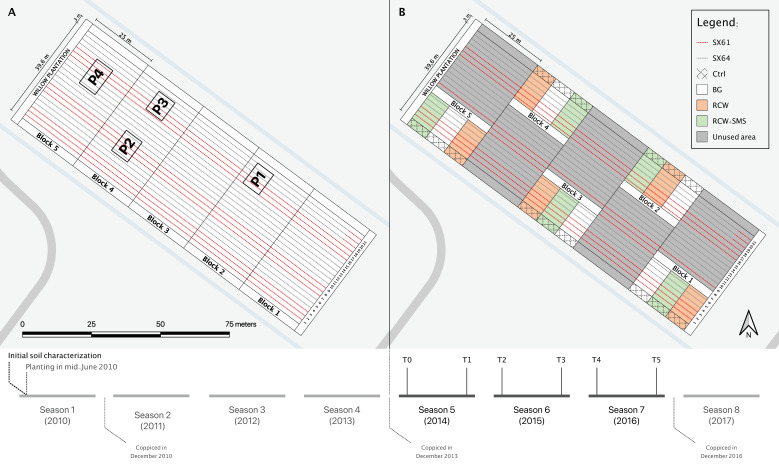
Evolution of the experimental design over time, including growth seasons, sampling times and coppicing times. (**A**) Experimental design of the first experimental phase, referred to as the GERLED site in Guidi et al. [55]. P1, P2, P3 and P4 were the sampling plots in their study; (**B**) Experimental design of the current experiment. T0 to T5 are the moments corresponding to the soil sampling. Adapted from Guidi et al. [55].

A willow plantation of 5475 m^2^ was established on the site in mid-June 2010. The plantation included two willow cultivars, ‘SX61’ and ‘SX64’ (*Salix miyabeana*), randomly planted in seven groups of three rows for a total of 21 rows (Figure 2A). The planting was carried out mechanically, following a SRIC technique [123]. Plants were spaced 1.8 × 0.3 m, at a density of 18,500 plants per hectare [55]. The plantation was coppiced for the first time in December 2010 and again three years later, in December 2013.

The experimental setup was modified in July 2014 for the purposes of the current study. The new setup included five blocks of 13.8 × 25 m comprising three rows of ‘SX61’; three rows of ‘SX64’; and a control zone without willow, hereafter referred to as Ctrl (Figure 2B). The Ctrl sections were located at the extremity of each block. Each section was split into three plots, to which three ground cover treatments were randomly applied: RCW (ramial chipped wood of *Salix* spp.); RCW+SMS (RCW mixed with spent mushroom substrate of *Pleurotus ostreatus*); and BG (bare ground). The RCW Agro Énergie, St-Roch-de-l’Achigan, Québec) was applied in a 10 cm thick layer. The SMS was obtained from a specialty mushroom farm (Advitam Inc., St-Ours, QC, Canada). A volume of 1.33 m^3^ of SMS was manually incorporated to the RCW in every RCW+SMS subsection, each of which measured 115 m^2^. Throughout the experiment, the plots without willows were mown and weeded to suppress vegetation using a string trimmer. The willow plantation was coppiced in December 2016 to end a third cycle of plant growth.

### 4.3. Soil Sampling

As shown in Figure 2A, soil samples were collected in June 2014 (T0) and at five other times over the course of the study: November 2014 (T1); June 2015 (T2); November 2015 (T3); June 2016 (T4); and November 2016 (T5). Each soil sample (one per treatment per block per time) was composed of three sub-samples collected at a depth of 0–30 cm. To limit the biases caused by the possible heterogeneous distribution of pollutants in the soil, each sample was collected within a 30 cm radius of its first corresponding sample (T0). Samples were placed in amber glass containers (System Plus Ltd., Baden, ON, Canada) and immediately sent to an external certified laboratory for chemical analysis (AGAT Laboratories Ltd., Montreal, QC, Canada) to assess the soil concentrations of polychlorinated biphenyls (PCBs) by GC-MS, petroleum hydrocarbons (C10-C50) by GC-FID, six TEs (i.e., Cd, Cr, Cu, Ni, Pb and Zn) by ICP-OES and polycyclic aromatic hydrocarbons (PAHs) by GC-MS, following the recommended provincial methods for environmental analyses [118,119,120,121,122]. Quality control and quality assurance were maintained for each method using duplicates, blanks and certified standard reference materials, following the provincial guidelines for analytical work in chemistry DR-12-SCA-01 [124]. Minimum frequency of insertion for blank and standard reference materials was one for each 10 samples. The recovery percentage for all standard reference materials, spiked blanks and fortified samples should be between 80% and 120% for all TEs and between 70% and 130% for C10-C50, PCBs and PAHs.

At T0, T1 and T5 only, extra soil samples were collected in RCW and BG subsections of ‘SX61’ and Ctrl sections, and put in 50 mL polypropylene tubes (Sarstedt Inc, Newton, NC, USA) to assess the soil trace element bioavailability according to a water extraction method described in Courchesne et al. [103] and in Séguin et al. [67]. Briefly, in a ratio of 1:10 soil to ultra-pure water, each sample was shaken for 2 h and centrifuged (1400 g) for 15 min. Aliquot of the unfiltered solution was used for pH and electrical conductivity (EC) analyses. The remaining solution was filtered using a 0.45 µm nylon membrane (nylon plain, product code 1213776, GVS Magna^TM^, Zola Predosa, BO, Italy). The water extracts were then acidified with 0.2% trace metal grade HNO_3_ and stored at 4 °C until TEs concentrations were measured by ICP-mass spectrometry (NexION^®^ 300, Perkin Elmer, Guelph, ON, Canada).

### 4.4. Biomass Sampling

Sampling of willow biomass was carried out after autumn leaf drop, in November 2016 (T5). In each plot, four plants of each cultivar (for a total of 120 samples) were cut at the base of the trunk, using a forest brush cutter. Fresh weight was assessed in the field using a parcel scale (GRAM SAFIR X50, Barcelona, Spain) and subsamples from each plant were brought to the laboratory, then oven dried at 105 °C to evaluate the percentage of moisture and estimate dry weight. These same subsamples were subsequently crushed and sent to an external laboratory (AGAT Laboratories Ltd., Montreal, QC, Canada) to analyze the TEs content (i.e., Cd, Cr, Cu, Ni, Pb and Zn) by ICP-OES following the recommended provincial methods for environmental analyses [121,122]. Quality control and quality assurance were maintained following the provincial guidelines for analytical work in chemistry DR-12-SCA-01 [124] as described for soil samples.

### 4.5. Data Analyses

Statistical analyses were performed using JMP^®^ Pro V.15.0.0 (SAS Institute, Cary, NC, USA). Raw data (soil contaminant concentrations at T0, biomass yield, biomass TEs concentrations and soil TEs bioavailability) were submitted to a two-way analysis of variance (ANOVA) test, followed by multiple comparisons of means according to Tukey’s Honestly Significant Difference (HSD) or simple comparisons of means according to Student’s *t*-test. Bioconcentration factor (BCF) was used to describe shoot accumulation relative to soil concentration. BCF was calculated by the following equation:

(1) $$\mathrm{BCF}=\frac{\;{\left[TE\right]}_{plant,i\;}}{{\left[TE\right]}_{soil,i}\;}$$

Given the significant differences in soil contaminant concentrations observed between raw values from planted and unplanted plots in the initial soil samples (T0) (see results), we decided to calculate the intermediate variation (IV) by plot at each subsequent sampling time (T1 to T5) to compare the treatment effects over time. The IV is the proportional variation of concentrations from a sampling time relative to the concentrations determined at T0. The IV was calculated by the equation:

(2) $${\mathrm{IV}}_{x}=\left(\frac{T_{x,i}-T_{0,i}}{T_{0,i}}\right)*100$$

where *x* is the subsequent sampling time (T1 to T5). The IV_T5_ can also be considered as the global variation (GV) over this study, since T5 refers to the final sampling time of the study. The IV was then submitted to a two-way ANOVA test, followed by multiple comparisons of means according to Tukey’s HSD. The IV values were mostly presented as the mean IV per treatment at each sampling time (T1 to T5), calculated by:

(3) $${\mathrm{Mean}\;\mathrm{IV}}_{x}=\frac{\sum_{i=1}^{n}\left(\frac{T_{x,i}-T_{0,i}}{T_{0,i}}\right)*100}{n}$$

where *x* is the subsequent sampling time (T1 to T5) and *n* represents each replicate.

The variation rate (VR) was also calculated by plot at each subsequent sampling time (T1 to T5) to compare the treatment effects over time in a different way. The VR is the proportional variation between a given time (T1 to T5) and its corresponding previous one. The VR was calculated by the equation:

(4) $${\mathrm{VR}}_{x}=\left(\frac{T_{x,i}-T_{x-1,i}}{T_{x-1,i}}\right)*100$$

where *x* is the subsequent sampling time (T1 to T5). The VR values were also submitted to the same statistical analyses as IV. Values were mostly presented as the mean VR per treatment at each subsequent sampling time (T1 to T5), obtained by:

(5) $${\mathrm{Mean}\;\mathrm{VR}}_{x}=\frac{\sum_{i=1}^{n}\left(\frac{T_{x,i}-T_{x-1,i}}{T_{x-1,i}}\right)*100}{n}$$

where *x* is the subsequent sampling time (T1 to T5) and *n* represent each replicate.

Period type has been added as a new two-level variable in the VR dataset in order to push the analysis further. The Growing level was attributed to VR values calculated over a growing period (*x* = T1, T3 and T5), while the Dormant level was attributed to VR values calculated over a dormant one (*x* = T2 and T4). The entire dataset of VR was then submitted to a three-way ANOVA test, followed by multiple comparisons of means according to Tukey’s HSD or simple comparisons of means according to Student’s *t*-test (when a period effect was observed) in order to compare values between ground cover treatments, cultivar treatments, and also by period type. The mean VR per treatment was calculated by period type and will referred as the mean seasonal variation (SV), which was calculated by the equation:

(6) $${\mathrm{Mean}\;\mathrm{SV}}_{p}=\frac{\sum_{i=1}^{n}VR_{p,i}}{n}$$

where *p* is the period (growing or dormant) and *n* represent each replicate.

Redundancy analysis (RDA) was performed on transformed variation factor (VF) values, using the vegan::rda function in R V.3.5.2 [125]. VF was calculated by the equation:

(7) $${\mathrm{VF}}_{x}=\frac{T_{x,i}}{T_{x-1,i}}$$

where *x* is the subsequent sampling time (T1 to T5). The VF data were normalized with a Hellinger transformation with the vegan::decostand function.

## 5. Conclusions

Two different cultivars of *Salix miyabeana* (‘SX61’ and ‘SX64’) supplemented with SMS of *Pleurotus ostreatus* and with RCW were tested in this study, with the goal of evaluating their impact on the in-soil variations of organic and inorganic contaminants over a period of three growing seasons. Results did not show that adding SMS as soil amendments improved the biomass yield of either cultivar or would have potential for remediating a mixed-contaminated soil in a cool temperate region. Although RCW did not show any effect on willow biomass yield, its application increased the soil concentrations of some PAHs over three growing seasons and improved the phytoextraction of Zn on average by 17.27%. This suggests that RCW may cause changes in mobility and bioavailability of some contaminants in soil. Therefore, RCW may be a promising soil amendment to reduce the leaching of PAHs in soil and to increase the efficiency of *Salix* spp. for rehabilitating zinc-contaminated sites.

After three seasons of growth, similar concentrations of Zn and Cu accumulated in the shoots of both cultivars, but ‘SX61’ showed slightly yet significantly higher concentrations of Cd than ‘SX64’. Both cultivars produced high biomass, up to 25 odt ha^−1^ yr^−1^, highlighting their great growth potential in moderately contaminated soils. This finding may also reflect the excellent agronomic conditions of the study site. In addition to meeting direct remediation needs, similarly contaminated land with good agronomic properties could be used for high biomass production of renewable feedstock for bioenergy and bioproducts.

Determining soil contaminant concentrations at the beginning of the study and then monitoring their relative variations over time revealed that ‘SX61’ significantly reduced the “natural attenuation” of C10-C50. For all other soil contaminants, GV were not significantly impacted by the presence of willows, despite evidence that some TEs were substantially eliminated from the ground by plant uptake. To a lesser extent, the presence of willows is suspected to have accentuated the increases of PCBs in soil after the three seasons of growth. At first glance, the GV reported in this study could appear to fall short of the remediation objectives that generally motivate the establishment of willow plantation on contaminated sites. However, considering the inconsistency between the extracted quantities of some TEs and their GV over time, these results could rather indicate the complexity of monitoring soil contamination in the field.

Throughout this study, willows were suspected of having acted as an evapotranspiration cover, thus reducing the leaching of contaminants into the environment, or even mobilizing some contaminants towards the rooting zones, especially when the transpiration rate was maximal. Findings supporting this are that many contaminants were subject to seasonal oscillations, showing average increases after a Growing period, yet lower increases or even decreases after a Dormant period. Such contaminant migration may have led to our inconclusive remediation results, even if degradation and/or phytoextraction processes would have been occurring simultaneously.

Our results highlight a phenomenon that certainly contributes to the difficulties of monitoring pollutant variations in the field over a long period of time, and that is rarely considered in phytoremediation studies. Based on the seasonal fluctuations reported here, we recommend comparing data from similar seasons, when monitoring soil contamination in the field, especially when soil is extensively penetrated by roots. Further investigation is underway in our laboratory to better understand the impact of SRIC of willow on the migration dynamics of soil contaminants.

## Figures and Tables

**Table 1:** Initial soil contaminant concentrations.

Parameters	Ctrl	SX61	SX64	*p*-Value
PCBs	26.72 ± 13.98 ^B^	83.69 ± 25.84 ^A^	45.28 ± 22.23 ^B^	0.006 **
C10-C50	1757.60 ± 1122.18 ^B^	6189.33 ± 2365.19 ^A^	3097.87 ± 1190.97 ^B^	<0.001 ***
Cadmium	1.78 ± 0.31 ^B^	2.23 ± 0.16 ^A^	1.91 ± 0.20 ^B^	0.019 *
Chromium	411.00 ± 200.10 ^C^	912.73 ± 143.32 ^A^	623.13 ± 157.63 ^B^	<0.001 ***
Copper	791.80 ± 382.98 ^B^	2381.33 ± 579.51 ^A^	1279.47 ± 559.65 ^B^	0.001 **
Nickel	66.20 ± 15.64 ^C^	106.47 ± 9.35 ^A^	84.93 ± 12.46 ^B^	<0.001 ***
Zinc	234.20 ± 75.27 ^C^	479.27 ± 54.00 ^A^	337.87 ± 66.96 ^B^	<0.001 ***
Acenaphthene	0.31 ± 0.21 ^B^	2.25 ± 3.75 ^A^	0.67 ± 0.25 ^B^	0.003 **
Acenaphtylene	1.71 ± 1.43 ^B^	8.63 ± 3.27 ^A^	4.25 ± 2.09 ^B^	<0.001 ***
Anthracene	6.00 ± 3.76 ^B^	34.96 ± 13.22 ^A^	19.50 ± 8.25 ^B^	0.001 **
Benz[a]anthracene	0.23 ± 0.16 ^B^	1.14 ± 0.54 ^A^	0.52 ± 0.25 ^B^	0.002 **
Benzo[a]pyrene	0.11 ± 0.08 ^B^	0.48 ± 0.25 ^A^	0.22 ± 0.11 ^B^	0.006 **
Benzo[ghi]perylene	0.12 ± 0.08 ^B^	0.71 ± 0.29 ^A^	0.36 ± 0.16 ^B^	0.002 **
Chrysene	0.18 ± 0.13 ^B^	0.57 ± 0.45 ^A^	0.25 ± 0.11 ^B^	0.004 **
Fluoranthene	0.23 ± 0.15 ^B^	1.40 ± 2.39 ^A^	0.40 ± 0.17 ^B^	0.004 **
Fluorene	0.41 ± 0.25 ^B^	2.14 ± 1.15 ^A^	0.98 ± 0.40 ^B^	0.004 **
Indeno[1,2,3-cd]pyrene	0.09 ± 0.07 ^B^	0.49 ± 0.19 ^A^	0.24 ± 0.12 ^B^	0.002 **
Naphthalene	0.19 ± 0.09 ^B^	0.65 ± 0.20 ^A^	0.34 ± 0.15 ^B^	<0.001 ***
Phenanthrene	1.01 ± 0.66 ^B^	3.36 ± 1.10 ^A^	1.79 ± 0.73 ^B^	<0.001 ***
Pyrene	0.51 ± 0.35 ^B^	2.99 ± 3.81 ^A^	1.06 ± 0.51 ^B^	0.006 **
1-Methylnaphthalene	0.23 ± 0.13 ^B^	0.61 ± 0.18 ^A^	0.35 ± 0.11 ^B^	<0.001 ***
2-Methylnaphthalene	0.23 ± 0.15 ^B^	0.62 ± 0.23 ^A^	0.40 ± 0.16 ^B^	0.002 **
1,3-Dimethylnaphthalene	0.29 ± 0.17 ^B^	0.87 ± 0.26 ^A^	0.48 ± 0.17 ^B^	<0.001 ***
2,3,5-Trimethylnaphthalene	0.09 ± 0.07 ^B^	0.61 ± 0.89 ^A^	0.20 ± 0.09 ^B^	0.001 **

Values are the averages (mean ± SD, *n* = 5 for Ctrl; *n* = 15 for ‘SX61’; *n* = 15 for ‘SX64’) of contaminant concentrations (mg kg^−1^), found in the initial soil samples (T0). Significance levels (*p*-value) are shown and asterisks (* *p* ≤ 0.05, ** *p* ≤ 0.01, *** *p* ≤ 0.001) indicate a significant difference in concentration between experimental conditions. Capital letters on the same row indicate differences according to Tukey’s HSD test.

**Table 2:** Global variation (GV) of soil PCBs, C10-C50 and six PAHs.

Treatments		PCBs	C10-C50	Benz[a]- anthracene	Benzo[ghi]- perylene	Naphthalene	1-Methyl- naphthalene	2-Methyl- naphthalene	1,3-Dimethyl- naphthalene
**Ctrl**	**BG**	23.87 ± 22.33	593.60 ± 643.07	0.14 ± 0.15	0.11 ± 0.11	0.08 ± 0.07	0.08 ± 0.07	0.08 ± 0.07	0.13 ± 0.15
		(−16.06 ± 53.42)	(−72.32 ± 12.30)	(+85.00 ± 343.97)	(+65.00 ± 244.69)	(−1.67 ± 168.68)	(−4.17 ± 170.12)	(−0.83 ± 168.85)	(+76.17 ± 348.86)
	**RCW**	27.40 ± 21.86	674.80 ± 402.98	0.27 ± 0.15	0.14 ± 0.11	0.14 ± 0.11	0.15 ± 0.07	0.11 ± 0.05	0.25 ± 0.21
		(+9.72 ± 53.16)	(−55.82 ± 26.21)	(+142.50 ± 248.05)	(+55.00 ± 144.05)	(+53.33 ± 250.32)	(+23.33 ± 157.83)	(−18.33 ± 74.16)	(+61.33 ± 176.24)
	**RCW+SMS**	33.19 ± 22.54	897.80 ± 803.55	0.20 ± 0.15	0.16 ± 0.15	0.11 ± 0.11	0.11 ± 0.11	0.11 ± 0.11	0.18 ± 0.19
		(+11.33 ± 38.73)	(−56.52 ± 18.15)	(+100.00 ± 335.88)	(+120.00 ± 325.19)	(+41.67 ± 256.24)	(+39.17 ± 257.72)	(+42.50 ± 256.18)	(+130.33 ± 431.78)
**SX61**	**BG**	103.84 ± 34.83	3742.00 ± 1327.96	0.86 ± 0.43	0.70 ± 0.42	0.36 ± 0.09	0.42 ± 0.13	0.38 ± 0.11	0.62 ± 0.08
		(+31.61 ± 9.56)	(−35.62 ± 14.07)	(−14.41 ± 27.35)	(−6.33 ± 27.24)	(−42.50 ± 17.22)	(−26.57 ± 30.23)	(−30.00 ± 36.13)	(−14.38 ± 35.33)
	**RCW**	89.78 ± 24.35	3528.00 ± 821.87	0.84 ± 0.25	0.70 ± 0.25	0.40 ± 0.07	0.44 ± 0.09	0.38 ± 0.13	0.60 ± 0.12
		(+29.81 ± 26.09)	(−29.38 ± 18.78)	(−9.74 ± 14.77)	(+12.30 ± 21.31)	(−27.05 ± 15.66)	(−17.52 ± 12.04)	(−27.67 ± 16.65)	(−24.13 ± 17.29)
	**RCW+SMS**	106.90 ± 46.26	3416.00 ± 1614.75	0.86 ± 0.40	0.64 ± 0.30	0.32 ± 0.08	0.36 ± 0.13	0.36 ± 0.09	0.42 ± 0.13
		(+4.45 ± 35.25)	(−48.95 ± 27.76)	(−21.08 ± 49.18)	(−3.33 ± 42.64)	(−51.90 ± 14.72)	(−41.27 ± 28.94)	(−42.93 ± 26.73)	(−51.92 ± 24.61)
**SX64**	**BG**	56.14 ± 22.32	1670.60 ± 583.58	0.40 ± 0.12	0.34 ± 0.09	0.22 ± 0.04	0.26 ± 0.09	0.20 ± 0.07	0.32 ± 0.13
		(+47.73 ± 44.60)	(−36.10 ± 12.04)	(−14.17 ± 42.25)	(−8.00 ± 25.15)	(−33.33 ± 20.41)	(−25.00 ± 34.36)	(−50.33 ± 29.59)	(−35.67 ± 38.90)
	**RCW**	61.20 ± 34.18	1831.80 ± 838.99	0.54 ± 0.19	0.40 ± 0.12	0.26 ± 0.05	0.28 ± 0.08	0.22 ± 0.04	0.32 ± 0.08
		(+30.77 ± 40.53)	(-49.81 ± 17.89)	(+281.56 ± 681.36)	(+138.67 ± 314.87)	(+66.90 ± 242.45)	(+104.00 ± 333.21)	(+15.71 ± 159.19)	(+99.90 ± 335.62)
	**RCW+SMS**	57.64 ± 24.17	1812.60 ± 955.62	0.44 ± 0.21	0.33 ± 0.17	0.19 ± 0.09	0.27 ± 0.13	0.23 ± 0.11	0.36 ± 0.18
		(+35.88 ± 26.77)	(-39.66 ± 18.54)	(−11.11 ± 43.21)	(−8.33 ± 45.64)	(−36.67 ± 21.73)	(−18.33 ± 39.70)	(−35.00 ± 28.50)	(−17.67 ± 27.73)
***p*-value**	**Cover**	0.782	0.835	0.057	0.038 *	0.035 *	0.026 *	0.059	0.045 *
	**Cultivar**	0.081	0.048 *	0.886	0.968	0.713	0.497	0.758	0.864
	Cover*Cultivar	0.305	0.216	0.796	0.530	0.830	0.741	0.820	0.475
**Interpretation**		-	**Ctrl ^B^** **SX61 ^A^** **SX64 ^AB^**	-	**BG ^B^** **RCW ^A^** **RCW+SMS ^AB^**	**BG ^B^** **RCW ^A^** **RCW+SMS ^AB^**	**BG ^B^** **RCW ^A^** **RCW+SMS ^B^**	-	**BG ^B^** **RCW ^A^** **RCW+SMS ^AB^**

The first value is the average (mean ± SD, *n* = 5) contaminant concentration (mg kg^−1^) for each treatment at T5. The value in parentheses, below the preceding one, represents the average (mean ± SD, *n* = 5) GV (%). Negative values indicate a decrease in concentration. Significance levels (*p*-value) are shown and asterisks (* *p* ≤ 0.05, ** *p* ≤ 0.01, *** *p* ≤ 0.001) indicate a significant effect of Cover, Cultivar or Cover*Cultivar on GV values. Capital letters in the final row were used to identify significant differences between treatments according to Tukey’s HSD test (*p* ≤ 0.05).

**Table 3:** Seasonal variation (SV) of soil PCBs, nickel and eight PAHs.

Treatments			PCBs	Nickel		Benz[a]- anthracene	Benzo[ghi]- perylene	Chrysene	Naphthalene	Phenanthrene	1-Methyl- naphthalene	1,3-Dimethyl- naphthalene	2,3,5-Trimethyl- naphthalene
**Growing period (Gr)**	**Ctrl**	**BG**	17.56 ± 75.04	−5.03 ± 20.14		47.44 ± 241.92	41.67 ± 185.57	92.39 ± 247.43	25.44 ± 190.28	266.32 ± 1007.36	28.33 ± 191.40	86.74 ± 344.78	25.00 ± 137.26
		**RCW**	40.08 ± 70.25	26.29 ± 71.85		100.00 ± 206.80	67.86 ± 111.99	136.31 ± 227.72	32.26 ± 143.34	689.22 ± 2424.26	41.92 ± 149.79	196.89 ± 669.04	80.00 ± 310.00
		**RCW+SMS**	18.31 ± 48.83	6.93 ± 26.79		7.74 ± 95.94	25.00 ± 89.34	42.86 ± 160.92	28.57 ± 197.01	219.93 ± 729.20	21.73 ± 149.75	80.12 ± 300.19	78.57 ± 307.89
	**SX61**	**BG**	34.60 ± 58.12	12.03 ± 29.59		4.05 ± 45.03	13.40 ± 37.15	20.06 ± 43.56	−5.87 ± 49.87	42.61 ± 94.61	−3.60 ± 60.83	19.66 ± 79.22	24.72 ± 83.46
		**RCW**	26.55 ± 29.69	12.92 ± 31.02		−1.67 ± 30.14	6.79 ± 23.46	17.33 ± 37.80	−14.97 ± 26.78	26.73 ± 106.34	−9.53 ± 37.08	6.40 ± 86.39	25.72 ± 163.51
		**RCW+SMS**	35.20 ± 56.89	10.38 ± 27.87		−5.03 ± 41.01	17.18 ± 52.78	0.17 ± 39.60	−16.63 ± 40.32	13.01 ± 93.42	−20.29 ± 38.47	−9.35 ± 66.81	4.18 ± 153.05
	**SX64**	**BG**	31.03 ± 96.53	13.97 ± 42.99		32.93 ± 186.42	36.22 ± 133.09	41.56 ± 140.34	−2.44 ± 87.40	24.82 ± 133.69	15.78 ± 138.34	14.66 ± 104.99	5.33 ± 102.32
		**RCW**	29.46 ± 27.87	9.28 ± 21.25		34.30 ± 134.01	29.44 ± 80.96	59.78 ± 136.71	1.25 ± 86.64	184.57 ± 697.84	9.03 ± 87.98	25.25 ± 138.93	−9.17 ± 51.57
		**RCW+SMS**	25.50 ± 31.96	8.04 ± 15.96		−1.89 ± 49.81	4.22 ± 33.77	15.52 ± 65.87	2.78 ± 47.52	49.85 ± 108.16	−6.48 ± 35.52	34.82 ± 113.74	15.83 ± 97.08
**Dormant period (Dr)**	**Ctrl**	**BG**	1.79 ± 57.36	0.77 ± 34.50		5.00 ± 47.96	−10.00 ± 21.08	−5.67 ± 75.63	1.67 ± 49.35	−13.93 ± 53.78	12.50 ± 90.71	15.12 ± 119.25	31.67 ± 103.77
		**RCW**	−17.85 ± 32.55	−23.63 ± 20.91		−0.93 ± 81.06	−17.59 ± 52.45	−30.56 ± 55.28	20.37 ± 106.65	7.53 ± 91.99	36.11 ± 116.07	13.05 ± 85.71	92.59 ± 137.97
		**RCW+SMS**	−14.54 ± 20.75	−16.68 ± 12.32		28.70 ± 111.89	−12.96 ± 20.03	15.37 ± 113.66	−3.70 ± 42.92	1.26 ± 102.21	18.33 ± 109.66	15.19 ± 77.05	57.41 ± 146.75
	**SX61**	**BG**	−11.23 ± 40.50	−13.06 ± 12.52		21.83 ± 95.71	−7.03 ± 41.41	8.65 ± 76.65	7.06 ± 61.05	5.49 ± 124.14	40.61 ± 132.28	39.63 ± 174.37	72.33 ± 236.07
		**RCW**	−13.98 ± 27.70	−14.78 ± 6.01		13.29 ± 52.95	5.71 ± 43.37	−11.08 ± 35.96	22.83 ± 53.98	7.45 ± 72.06	50.83 ± 105.30	32.56 ± 95.22	31.19 ± 88.35
		**RCW+SMS**	−23.21 ± 34.42	−17.33 ± 7.16		14.63 ± 63.10	−11.05 ± 32.58	−2.24 ± 28.35	14.67 ± 50.67	13.85 ± 76.20	62.67 ± 145.55	38.70 ± 112.60	73.25 ± 148.85
	**SX64**	**BG**	18.76 ± 69.77	−14.73 ± 19.55		18.33 ± 81.78	−2.50 ± 52.42	3.21 ± 64.43	30.00 ± 69.30	12.57 ± 55.10	26.00 ± 73.41	14.83 ± 56.73	60.00 ± 90.68
		**RCW**	−16.39 ± 34.73	−16.30 ± 6.27		9.52 ± 34.95	−1.50 ± 20.07	−11.17 ± 33.19	34.17 ± 66.72	5.41 ± 74.33	31.67 ± 95.65	16.96 ± 56.88	40.00 ± 80.97
		**RCW+SMS**	−3.64 ± 61.50	−14.07 ± 6.92		61.00 ± 203.73	−1.67 ± 38.05	9.50 ± 88.81	−6.67 ± 43.18	0.94 ± 87.90	36.67 ± 120.20	5.11 ± 93.11	92.62 ± 239.59
***p*-value**		**Cover**	0.571	0.681		0.002 **	0.028 *	0.042 *	0.021 *	0.015 *	0.006 **	0.005 **	0.405
		**Cultivar**	0.024 *	0.072		0.865	0.924	0.602	0.589	0.854	0.384	0.770	0.682
		**Period**	<0.001 ***	0.024 *		0.641	0.007 **	0.009 **	0.028 *	0.012 *	0.025 *	0.850	0.015 *
		Cover*Cultivar	0.165	0.767		0.846	0.890	0.698	0.919	0.842	0.755	0.825	0.402
		Cover*Period	0.255	0.207		0.412	0.635	0.491	0.811	0.998	0.853	0.912	0.941
		Cultivar*Period	0.725	0.994		0.838	0.726	0.253	0.890	0.655	0.803	0.825	0.817
		Cover*Cultivar*Period	0.601	0.013 *		0.589	0.214	0.855	0.549	0.562	0.970	0.560	0.549
**Interpretation**			**Ctrl ^B^** **SX61 ^AB^** **SX64 ^A^**	**Gr::Ctrl**	**BG ^B^** **RCW ^A^** **RCW+SMS ^AB^**	**BG ^B^** **RCW ^A^** **RCW+SMS ^B^**	**BG ^B^** **RCW ^A^** **RCW+SMS ^AB^**	**BG ^AB^** **RCW ^A^** **RCW+SMS ^B^**	**BG ^B^** **RCW ^A^** **RCW+SMS ^B^**	**BG ^B^** **RCW ^A^** **RCW+SMS ^B^**	**BG ^B^** **RCW ^A^** **RCW+SMS ^B^**	**BG ^B^** **RCW ^A^** **RCW+SMS ^B^**	-
			**Gr > Dr**	**Gr > Dr**		-	**Gr > Dr**	**Gr > Dr**	**Gr < Dr**	**Gr > Dr**	**Gr < Dr**	-	**Gr < Dr**

Values are the mean SV_p_ (mean ± SD) (%) for each treatment, observed either after a Growing period (Gr, *n* = 15 for each treatment) or after a Dormant period (Dr, *n* = 10 for each treatment). Negative values indicate a decrease in concentration. Significance levels (*p*-value) are shown and asterisks (* *p* ≤ 0.05, ** *p* ≤ 0.01, *** *p* ≤ 0.001) indicate a significant effect of Cover, Cultivar, Period, Cover*Cultivar, Cover*Period, Cultivar*Period and Cover*Cultivar*Period on SV. Capital letters and symbols of comparison (> or <) in the final row were used to identify significant differences between treatments according to Tukey’s HSD test (*p* ≤ 0.05).

**Table 4:** Mean water extracted concentration of TEs, pH and EC in soil over time.

Parameters	Times	Units	Ctrl		SX61			*p*-Value		Interpretation
			BG	RCW	BG	RCW	Cover	Cultivar	Cover * Cultivar	
pH	T0	-	7.68 ± 0.30	-	7.89 ± 0.09	-	-	0.133	-	-
EC		µS cm^−1^	127.50 ± 37.96	-	120.45 ± 9.09	-	-	0.675	-	-
Chromium		mg kg^−1^	0.24 ± 0.15	-	0.33 ± 0.05	-	-	0.252	-	-
Copper		mg kg^−1^	3.09 ± 1.84	-	4.06 ± 1.43	-	-	0.516	-	-
Nickel		mg kg^−1^	0.04 ± 0.02	-	0.03 ± 0.00	-	-	0.102	-	-
Zinc		mg kg^−1^	0.14 ± 0.01	-	0.15 ± 0.02	-	-	0.384	-	-
pH	T1	-	7.90 ± 0.24	7.91 ± 0.12	7.94 ± 0.13	7.92 ± 0.09	0.922	0.616	0.604	-
EC		µS cm^−1^	47.02 ± 10.91	91.09 ± 33.67	68.57 ± 11.74	74.14 ± 18.91	0.070	0.637	0.061	-
Chromium		mg kg^−1^	0.10 ± 0.04	0.27 ± 0.09	0.31 ± 0.06	0.32 ± 0.07	0.039 *	0.009 **	0.040 *	Ctrl:(BG < RCW) and BG:(Ctrl < SX61)
Copper		mg kg^−1^	1.41 ± 0.50	2.80 ± 1.44	3.88 ± 1.58	3.75 ± 0.51	0.162	0.047 *	0.095	Ctrl < SX61
Nickel		mg kg^−1^	0.03 ± 0.01	0.05 ± 0.01	0.03 ± 0.01	0.03 ± 0.01	0.147	0.011 *	0.289	Ctrl > SX61
Zinc		mg kg^−1^	0.12 ± 0.01	0.14 ± 0.01	0.14 ± 0.01	0.14 ± 0.01	0.070	0.216	0.094	-
pH	T5	-	7.91 ± 0.26	8.00 ± 0.05	8.01 ± 0.07	8.02 ± 0.07	0.381	0.277	0.552	-
EC		µS cm^−1^	47.56 ± 17.81	72.58 ± 39.63	67.72 ± 7.20	76.34 ± 16.76	0.071	0.271	0.269	-
Chromium		mg kg^−1^	0.15 ± 0.06	0.24 ± 0.14	0.33 ± 0.14	0.32 ± 0.08	0.012 *	0.008 **	0.237	BG<RCW and Ctrl < SX61
Copper		mg kg^−1^	1.78 ± 0.70	1.92 ± 0.77	4.08 ± 1.08	3.77 ± 0.54	0.684	0.026 *	0.305	Ctrl < SX61
Nickel		mg kg^−1^	0.04 ± 0.01	0.04 ± 0.01	0.03 ± 0.00	0.03 ± 0.01	0.373	0.008 **	0.748	Ctrl > SX61
Zinc		mg kg^−1^	0.12 ± 0.01	0.12 ± 0.01	0.13 ± 0.01	0.13 ± 0.02	0.936	0.039 *	0.987	Ctrl < SX61

Values are the average (mean ± SD, *n* = 5) concentrations (mg kg^−1^) of water extracted TEs by treatment at T0, T1 and T5. Significance levels (*p*-value) are shown and asterisks (* *p* ≤ 0.05, ** *p* ≤ 0.01, *** *p* ≤ 0.001) indicate a significant effect of Cover, Cultivar and Cover*Cultivar. Symbols of comparison (> or <) in the final column were used to identify significant differences between treatments according to Student’s *t*-test (*p* ≤ 0.05).

**Table 5:** Biomass parameters of willows after three seasons.

Parameters	Units		SX61			SX64			*p*-Value		Interpretation
		BG	RCW	RCW + SMS	BG	RCW	RCW + SMS	Cover	Cultivar	Cover * Cultivar	
Biomass	odt ha^−1^ yr^−1^	22.75 ± 7.86	26.35 ± 9.61	26.20 ± 12.89	28.83 ± 12.22	26.36 ± 9.89	28.38 ± 11.97	0.646	0.301	0.368	-
Humidity	%	55.85 ± 1.00	56.26 ± 1.00	55.44 ± 1.00	53.94 ± 1.00	53.84 ± 1.00	53.98 ± 1.00	0.387	<0.001 ***	0.076	SX61 ^A^, SX64 ^B^
Shoot [Cd]	mg kg^−1^	2.24 ± 0.00	2.42 ± 0.00	2.50 ± 1.00	2.02 ± 1.00	2.30 ± 1.00	2.36 ± 1.00	0.526	0.024 *	0.844	SX61 ^A^, SX64 ^B^
Shoot [Cu]		5.60 ± 1.00	5.60 ± 1.00	5.60 ± 1.00	5.00 ± 0.00	5.20 ± 1.00	5.20 ± 1.00	0.922	0.311	0.873	-
Shoot [Zn]		64.80 ± 4.00	78.20 ± 8.00	75.60 ± 11.00	68.60 ± 4.00	81.40 ± 13.00	77.60 ± 8.00	0.010 **	0.312	0.921	BG ^B^, RCW ^A^, RCW+SMS ^A^
Cd extraction yield	mg ha^−1^ yr^−1^	51.42 ± 16.71	63.22 ± 15.44	67.16 ± 33.96	61.25 ± 31.79	59.51 ± 18.58	69.07 ± 30.56	0.368	0.685	0.288	-
Cu extraction yield		129.46 ± 41.98	149.38 ± 47.13	148.88 ± 61.75	144.15 ± 33.14	133.82 ± 34.27	145.75 ± 29.23	0.613	0.953	0.430	-
Zn extraction yield		1470.03 ± 247.55	2035.75 ± 391.30	1956.97 ± 654.48	1996.43 ± 553.11	2114.64 ± 598.14	2198.83 ± 492.27	0.027 *	0.195	0.244	BG ^B^, RCW ^A^, RCW+SMS ^A^
BCF of Cd	Factor	1.04 ± 0.19	1.09 ± 0.15	1.07 ± 0.20	1.11 ± 0.36	1.13 ± 0.20	1.27 ± 0.33	0.699	0.089	0.299	-
BCF of Cu		0.0027 ± 0.0037	0.0028 ± 0.0021	0.0021 ± 0.0028	0.0048 ± 0.0013	0.0047 ± 0.0005	0.0056 ± 0.0005	0.893	0.079	0.207	-
BCF of Zn		0.14 ± 0.03	0.17 ± 0.02	0.15 ± 0.02	0.22 ± 0.05	0.23 ± 0.04	0.26 ± 0.12	0.711	0.028 *	0.407	SX61 ^B^, SX64 ^A^

Values are the averages (mean ± SD, *n* = 20 for biomass and humidity parameters; n=5 for all other parameters concerning TE) for each treatment. Significance levels (*p*-value) are shown and asterisks (* *p* ≤ 0.05, ** *p* ≤ 0.01, *** *p* ≤ 0.001) indicate a significant effect of Cover, Cultivar and Cover*Cultivar. Capital letters in the final column were used to identify significant differences between treatments according to Tukey’s HSD test (*p* ≤ 0.05).

**Table 6:** Soil characteristics of the site.

Parameters	Units	Values	Parameters	Units	Values
Cation-exchange capacity	meq 100g^−1^	43.50	PCBs ^c^	mg kg^−1^	57.58 ± 11.70
pH ^a^	-	7.70	Cadmium ^c^	mg kg^−1^	1.75 ± 0.15
pH buffer	-	>7.50	Chromium ^c^	mg kg^−1^	659.50 ± 127.22
Soil texture	-	Clay	Copper ^c^	mg kg^−1^	1380.00 ± 201.57
Clay	%	46.00	Nickel ^c^	mg kg^−1^	42.90 ± 2.22
Silt	%	33.90	Lead ^c^	mg kg^−1^	34.00 ± 8.12
Sand	%	20.10	Zinc ^c^	mg kg^−1^	386.50 ± 72.13
Organic matter	%	9.60	Acenaphthene ^c^	mg kg^−1^	0.56 ± 0.18
K+ Mg + Ca saturation	%	100.00	Acenaphtylene ^c^	mg kg^−1^	1.98 ± 0.38
P (P/Al) saturation	%	16.50	Anthracene ^c^	mg kg^−1^	18.15 ± 4.90
Ca saturation	%	81.60	Benz[a]anthracene ^c^	mg kg^−1^	0.43 ± 0.09
K saturation	%	3.10	Benzo[a]pyrene ^c^	mg kg^−1^	0.28 ± 0.07
Mg saturation	%	15.30	Benzo[ghi]perylene ^c^	mg kg^−1^	0.48 ± 0.12
**Parameters**	**Units**	**Values**	Chrysene ^c^	mg kg^−1^	0.40 ± 0.09
Ca ^b^	mg kg^−1^	7090.00	Fluoranthene ^c^	mg kg^−1^	0.54 ± 0.20
P ^b^	mg kg^−1^	80.00	Fluorene ^c^	mg kg^−1^	0.94 ± 0.21
K ^b^	mg kg^−1^	525.00	Indeno[1,2,3-cd]pyrene ^c^	mg kg^−1^	0.32 ± 0.09
Mg ^b^	mg kg^−1^	800.00	Naphthalene ^c^	mg kg^−1^	0.42 ± 0.13
Al ^b^	mg kg^−1^	48.00	Phenanthrene ^c^	mg kg^−1^	2.62 ± 0.71
Zn ^b^	mg kg^−1^	85.60	Pyrene ^c^	mg kg^−1^	1.34 ± 0.41
Cu ^b^	mg kg^−1^	417.00	1-Methylnaphthalene ^c^	mg kg^−1^	0.42 ± 0.13
Mn ^b^	mg kg^−1^	11.00	2-Methylnaphthalene ^c^	mg kg^−1^	0.42 ± 0.12
B ^b^	mg kg^−1^	1.40	1,3-Dimethylnaphthalene ^c^	mg kg^−1^	0.55 ± 0.18
Fe ^b^	mg kg^−1^	178.00	2,3,5-Trimethylnaphthalene ^c^	mg kg^−1^	0.40 ± 0.13

Soil samples were collected at 0-30 cm below ground. ^a^ Water extraction. ^b^ Melich III method. ^c^ Chemical analysis was performed by AGAT Laboratories Ltd. (Montreal, QC, Canada) following the recommended provincial methods for environmental analyses [118,119,120,121,122]. Five (5) soil samples were collected at 0–30 cm below ground in each plot (P1, P2, P3 and P4, see Figure 2A). Values are averages (mean ± SD, *n* = 20). The table was adapted from Guidi et al. [55].

## Data Availability

Not applicable.

## Supplementary Materials

The following are available online at https://www.mdpi.com/2223-7747/10/3/520/s1, Figure S1: Visual distribution of contaminant concentrations found in the initial soil samples and in those from the 2010 soil characterization, Table S1: Mean intermediate variations of soil contaminant concentrations by treatment at each subsequent sampling time, Table S2: Mean variation rates of soil contaminant concentrations by treatment at each subsequent sampling time. Table S3: Seasonal variation (SV) of soil PCBs, C10-C50, PAHs and TEs.
