# Supplementary material for: Short Rotation Intensive Culture of Willow, Spent Mushroom Substrate and Ramial Chipped Wood for Bioremediation of a Contaminated Site Used for Land Farming Activities of a Former Petrochemical Plant
Source: Plants (Basel). 2021 Mar 10;10(3):520. doi: 10.3390/plants10030520 (PMC7999893; doi:10.3390/plants10030520)
Supplement: Supplementary file 1 [file plants-10-00520-s001.zip › plants-1116697-supplementary-proof/Supplementary_Figure S1.docx]

**Figure S1.**Visual distribution of contaminant concentrations found in the initial soil samples and in those from the 2010 soil characterization. The box plots display the distribution of contaminant concentrations (mg kg^-1^) by sample group (n=5 for Ctrl; n=15 for ‘SX61’; n=15 for ‘SX64’ and n=20 for 2010 characterization). In each plot, the box boundaries represent the 25^th^ and 75^th^ percentiles, the horizontal thin black line represents the median and the red diamond symbol refers to the mean. The whiskers represent ﻿1.5 times the interquartile range of the distribution. The outliers are denoted as larger points outside the whiskers.
