# Supplementary figures and images for: Short Rotation Intensive Culture of Willow, Spent Mushroom Substrate and Ramial Chipped Wood for Bioremediation of a Contaminated Site Used for Land Farming Activities of a Former Petrochemical Plant
Source: Plants (Basel). 2021 Mar 10;10(3):520. doi: 10.3390/plants10030520 (PMC7999893; doi:10.3390/plants10030520)

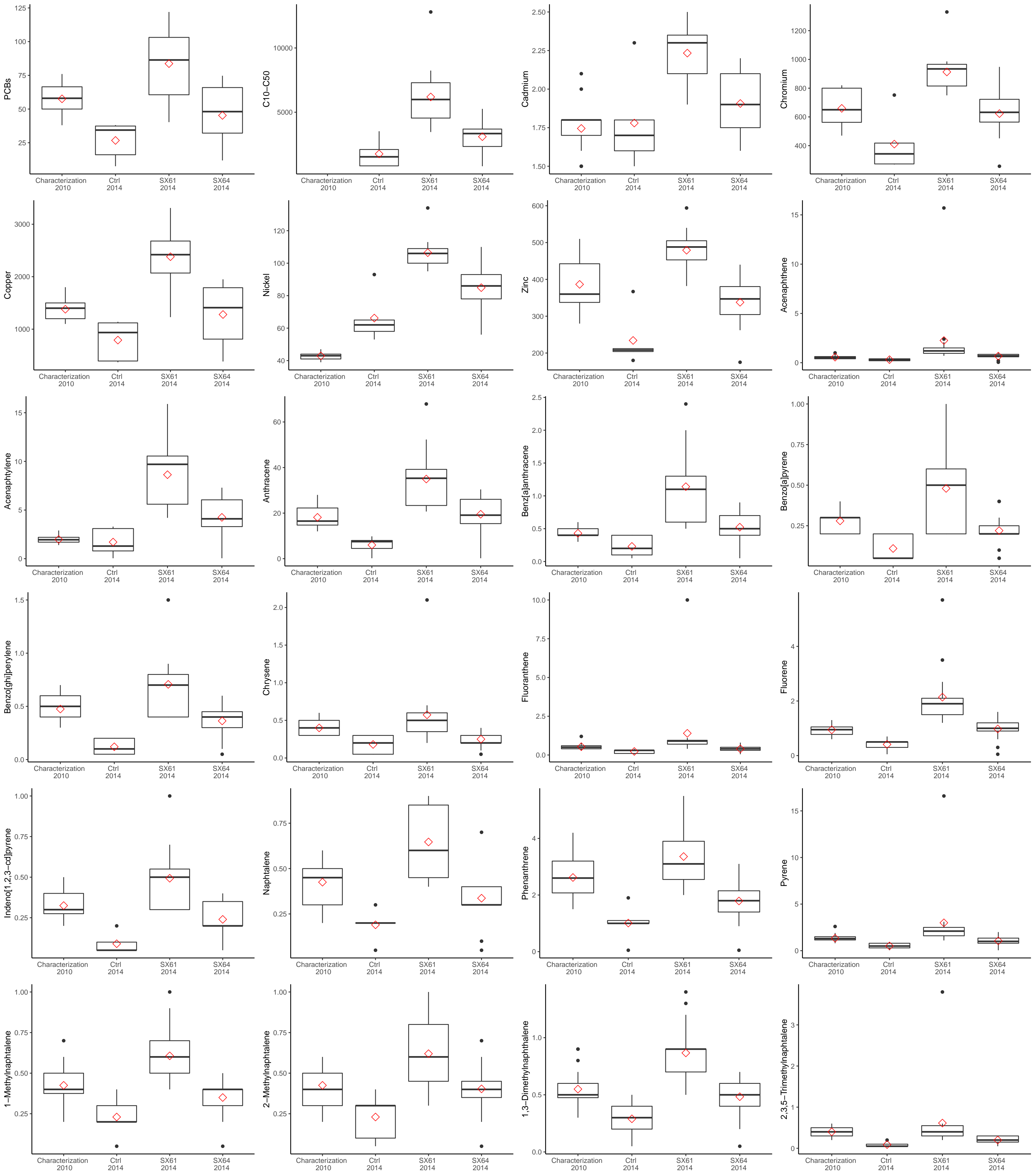

Supplement: Supplementary file 1 [file plants-10-00520-s001.zip › plants-1116697-supplementary-proof/Figure S1.pdf]
